# Engineering of W-shaped benzodithiophenedione-based small molecular acceptors with improved optoelectronic properties for high efficiency organic solar cells

**DOI:** 10.1039/d2ra03280e

**Published:** 2022-08-08

**Authors:** Ehsan Ullah Rashid, N. M. A. Hadia, Javed Iqbal, Rana Farhat Mehmood, H. H. Somaily, Sahar Javaid Akram, Ahmed M. Shawky, Muhammad Imran Khan, Sadia Noor, Rasheed Ahmad Khera

**Affiliations:** Department of Chemistry, University of Agriculture Faisalabad 38000 Pakistan javedkhattak79@gmail.com javed.iqbal@uaf.edu.pk rasheedahmadkhera@yahoo.com rasheed.ahmad.khera@uaf.edu.pk; Physics Department, College of Science, Jouf University Sakaka Al-Jouf P. O. Box 2014 Saudi Arabia nmhadia@ju.edu.sa; Department of Chemistry, Division of Science and Technology, University of Education Township Lahore 54770 Pakistan; Research Center for Advanced Materials Science (RCAMS), King Khalid University Abha 61413 P.O. Box 9004 Saudi Arabia; Department of Physics, Faculty of Science, King Khalid University Abha P.O. Box 9004 Saudi Arabia; Science and Technology Unit (STU), Umm Al-Qura University Makkah 21955 Saudi Arabia

## Abstract

In the current study, with the objective to improve the overall performance of organic solar cells, seven new W-shaped small molecular acceptors – were developed theoretically by the end-group alteration of the reference (WR) molecule. The MPW1PW91 functional with the basis set 6-31G(d,p) was used to explore the optoelectronic properties of the WR and W1–W7 molecules and the time-dependent self-consistent filed (TD-SCF) simulation was used to investigate the solvent-state calculations. The several explored photovoltaic attributes were the absorption spectra, excitation energies, bandgap between the FMOs, oscillator strength, full width at half maximum, light-harvesting efficiency, transition density matrices, open-circuit voltage, fill factor, density of states, binding energy, interaction coefficient, *etc.* Overall, the results revealed a bathochromic shift in the absorption maxima (*λ*_max_), a reduced HOMO–LUMO gap (*E*_gap_), and smaller excitation energy (*E*_x_) of the altered molecules as compared to the WR molecule. Some of the optoelectronic aspects of a well-known fused ring based acceptor named Y6 are also compared with the studied W-shaped molecules. Additionally, the W1 molecule presented the smallest *E*_gap_, along with highest *λ*_max_ and the lowest *E*_x_, amongst all, in both the evaluated media (gas and solvent). The open circuit voltage (*V*_OC_) of all the considered small molecular acceptors was calculated by pairing them with the PTB7-Th donor. Here, W6 and W7 displayed the best results for the *V*_OC_ (1.48 eV and 1.51 eV), normalized *V*_OC_ (57.25 and 58.41) and FF (0.9131 and 0.9144). Consequently, in light of the results of this research, the altered molecules could be considered for practical implementation in the manufacturing of OSCs with improved photovoltaic capabilities.

## Abstract 2

The developed molecules have a reduced band gap and lower excitation energy. Their *V*_OC_ was calculated by making complexes of them with the PTB7-Th donor.

## Graphical abstract

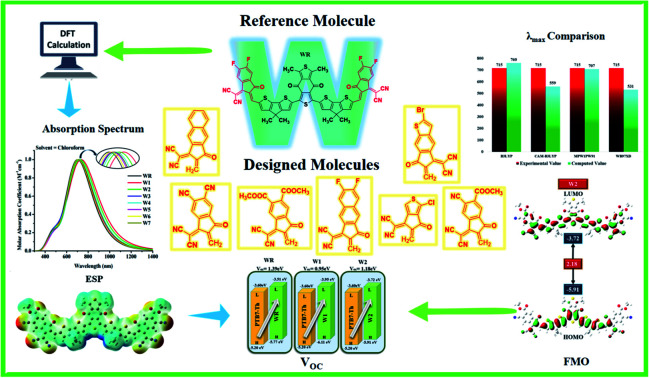

## 1 Introduction

Organic solar cells (OSCs) are light weight, reasonably priced, mechanically flexible, semi-transparent, and easy to fabricate. They also hold considerable potential in advancing the achievement of carbon-neutral energy.^1–6^ Due to substantial advancements in materials research, mechanics, and device fabrication, the power conversion efficiency (PCE) of organic photovoltaic cells (OPVs) has increased from 11% to 19% in the previous five years.^7^ Although fullerene-based OSCs have dominated the photovoltaic market for more than 20 years, their dominance has been limited owing to a number of negative features, including poor absorption in the UV-visible range, reduced tunability of energy levels, and high prices.^8^ Thus, the steady rise in the PCEs of OPVs over the past five years could be due to the endless expansion of non-fullerene acceptors (NFAs). This increase in solar efficiency of NFA-based photovoltaic cells is attributed to their greater light-harvesting efficiency, wide absorption in the UV-visible range, and tunable energy level, among various other advantages.^2,9–12^

A prominent class of NFAs, namely the fused-ring electron acceptors (FREAs), were successful in bringing OSCs to an upgraded level of performance.^13–16^ The fused-ring core in the molecular skeleton of the FREAs is the most noticeable structural feature of their structure.^1^ Poly-heterocyclic fused-ring units, *i.e.*, indacenodithiophene (IDT),^17–19^ indacenodithieno[3,2-*b*]thiophene (IDTT),^13,20^ thienothiophen[3,2-*b*]-pyrrolobenzothiadiazole,^14,15,21,22^ or thienothiophen[3,2-*b*]-pyrrolo benzotriazole^23–25^ are known to be the key to the revolutionary architectural engineering of acceptor–donor–acceptor (A–D–A) or A–D–A–D–A structural designs. Extending central core diameters from fused five-member rings to fused nine-member rings, or even more complicated fusion heterocycles, efficiently regulates the optoelectronic characteristics of FREAs, which produce exceptional photovoltaic performance.^26–30^ But these complicated fusion rings lead to the problem of high synthetic costs and hazardous chemicals utilization, making them impractical for large-scale commercial use in the near future.^31^

So, NFAs with a shortened structural configuration, as well as simplified fabrication procedures with mildly hazardous chemicals are indisputably more significant.^32^ These non-fullerene acceptors based exclusively on two to three heterocycle-based structures may be regarded as simple fused-ring acceptors (SFAs).^33^ The SFA molecules of the NFAs class of OPVs have recently attracted a lot of interest.^34^ Scientists have synthesized benzodithiophenedione (BDD) based small SFA molecules (BDDEH-4F and BDDBO-4F) by a straight heteroarylation approach free from ligands. As compared to the formal approaches towards the synthesis, only three steps were employed for the synthesis of these small acceptor molecules. The significance of the BDD core can be estimated by the fact that the well-known polymer donors PM6 ref. 35 and PM7 ref. 36 for efficient polymer solar cells also include this component. These A2–D–A1–D–A2 structure based BDDEH-4F and BDDBO-4F molecules contain 2-ethylhexyl and 2-butyloctyl alkyl-chains, respectively, on their BDD core. The intermolecular non-covalent attractions between the oxygen atoms of the carbonyl group and the sulphur atoms of the cyclopentadithiophene (CPDT) units allow BDDEH-4F and BDDBO-4F to keep geometry in single plan. Moreover, their optical and electrochemical characteristics are seen to be almost unaffected by the varying alkyl-chain sizes on their BDD core.^32^

**Fig. 1:**
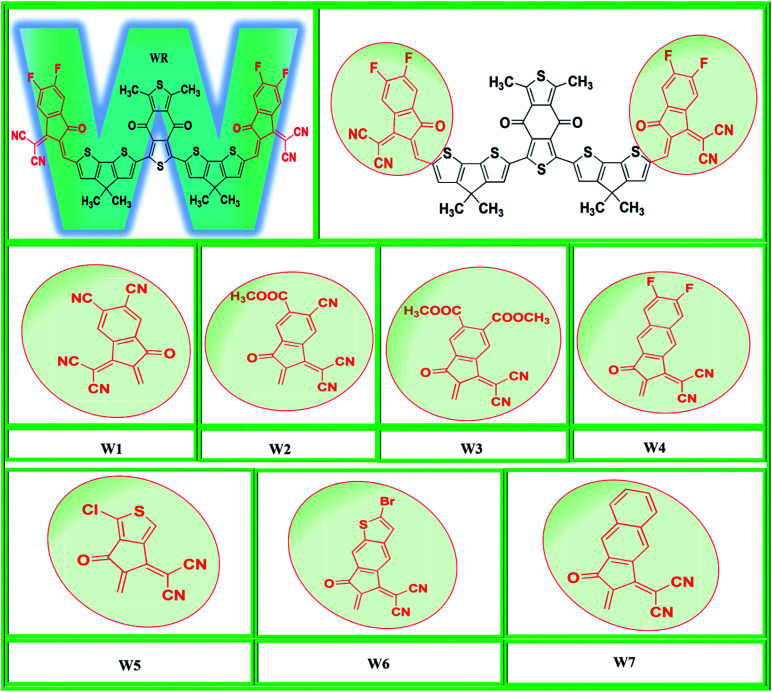
Molecular designing scheme of W1–W7 molecules from WR reference molecule.

In the present computational approach, the basic molecular structure on which the above two mentioned molecules are based (BDDEH-4F and BDDBO-4F) is taken as reference molecule (WR) with the only difference of methyl groups in the places of alkyl long chains because of their significantly low influence on the optoelectronic properties of molecules. For instance, the difference in the maximum absorption wavelength of both these molecules in the absorption spectrum was of only 1 nm.^32^ So, from the framework of these molecules, we have developed seven new W-shaped acceptor molecules (W1–W7) of A2-D-A1-D-A2 types through end-capped engineering, *i.e.*, substituting the existing terminal groups with some innovative electron withdrawing groups. Y6, a very widely reported acceptor molecule with fused-ring based core possesses some excellent optoelectronic properties such as smaller *E*_gap_, excellent charges mobility and exciton binding energy *etc*.^14^ Our W-shaped newly developed have structural similarities with Y6, so in this study we have also compared some of the optoelectronic properties of Y6 with newly proposed molecule. The molecular designing scheme of this end-capped modification is shown in Fig. 1.

## 2 Computational methodolgy

The density functional theory (DFT) technique is the new standard for quantum mechanical research in the computing domain.^37,38^ For this reason, Gaussian 09^39^ was used to perform geometric calculations on all molecules, and GaussView 6.0.16 ref. 40 was used to create and view all the three dimensional molecular assemblies. As a first step, at 6-31G(d,p) basis set and with a restricted spin, four exchange-correlation functionals, B3LYP,^41^ CAM-B3LYP,^42^ MPW1PW91,^43^ and ωB97XD,^44^ were used to optimize the structure of reference (WR). The reason behind the restriction of spin was to avoid any possible spin contamination during computation. Then, through proper evaluation the most favorable one out of all these functionals was selected to carry out further analysis. This selection of functional was carried out through the comparison of the absorption spectra attained from the time-dependent self-consistent field (TD-SCF) simulations in the gaseous form, as well as the solvent (chloroform) state with the experimental one of WR.^45^ Here, the influence of solvent (Chloroform) was studied with the use of polarizable continuum model (PCM) of integral equation formalism (IEFPCM).^46^

After the comparative analysis of computational *λ*_max_ with the experimental value it was estimated that Modified Perdew-Wang 1-parameter (MPW1PW91) functional possess the closest coherence with the cited *λ*_max_, which gave us a good indicator that this functional in combination with the designated basis set would work well for calculating the photovoltaic attributes of the altered molecules.^32^ At this point, the SWizard^47^ program was used to process the absorption maximum results, and in order to display the spectral depiction, Origin 6.0 ref. 48 application was utilized. Multiwfn-software^49^ was used to turn transition density matrix data into maps showing exciton interactions and movements. Furthermore, PyMOlyze 1.1 software^50^ was used to examine the role of different fragments (donor, acceptor1 and acceptor2) of the molecules in the density of states (DOS)^51^ of WR and W1–W7 molecules.

Reorganization energy (RE) is an important parameter for the determination of the amount of charge movements, *i.e.*, intermolecular or intramolecular, which can be analyzed by using Marcus theory.^52,53^ However, the intramolecular charge transfer (ICT) phenomena is the primary focus of our research. Actually, the external and internal reorganization energies are combined to form the overall RE. While, a sudden change in the outer atmosphere, along with the polarization changes during charge transfer are some of the examples of the external RE, the internal RE involves variance in molecular structures. Due to inability of external RE to support our computations because environmental conditions have such a huge role in determining the amount of external reorganization energy, a precise computational calculation is impossible for external RE.^54^ We exclusively focused on internal RE in this study. The mobility of cations (*λ*_+_) and anions (λ_−_), which collectively constitute the internal RE, was measured using the given eqn (1) and (2),^54^ which are centered on the DFT-based functional MPW1PW91/6-31G(d,p).

1 *λ*_+_ = [*E*^0^_+_ − *E*_0_] + [*E*_0_^+^ − *E*_+_]

2 *λ*_−_ = [*E*^0^_−_ − *E*_0_]+[*E*_0_− − *E*_−_]

Optimized neutral molecular geometries resulting in cation and anion energies are *E*_0_^+^ and *E*_0_−, respectively. *E*_−_ and *E*_+_, on the other hand, are optimized geometries of anions and cations, respectively.*E*^0^_−_ and *E*^0^_+_ are the energies of neutral molecules, which are determined using optimal anion and cation structures. Lastly, the optimized neutral molecule's single point charge in the ground state is written as *E*_0_.^55^

## 3 Results and discussion

### 3.1. Chemistry of molecules

**Fig. 2:**
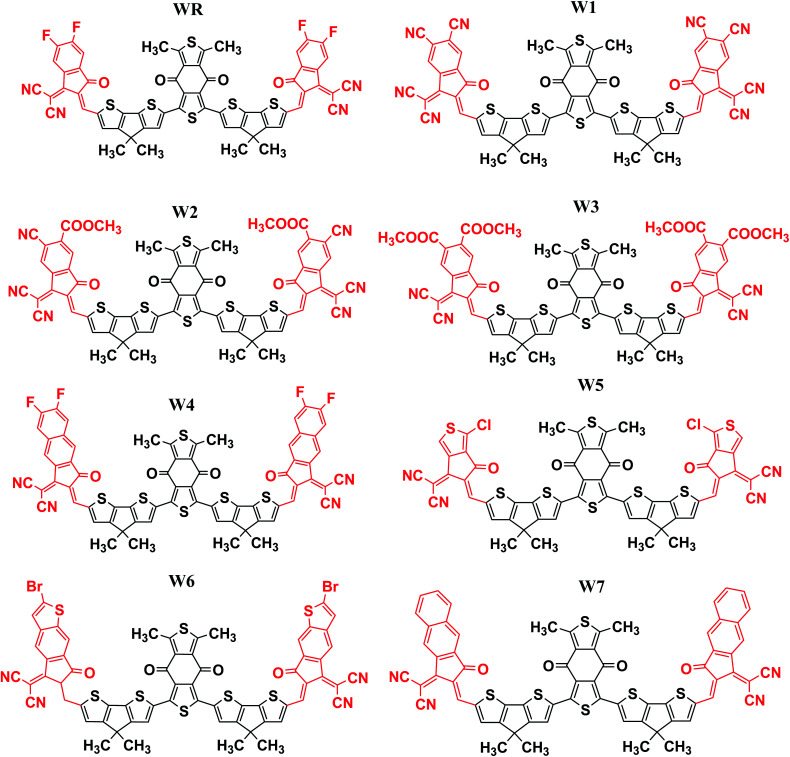
ChemDraw structures of WR and W1–W7 molecules.

In this research, we have considered WR as our reference molecule, which contain 1,3-dimethyl-2,6-dithia-s-indacene-4,8-dione as the central acceptor unit (A1) that is further attached to 4,4-dimethyl-4*H*-cyclopenta[2,1-*b*;3,4-*b*′]dithiophene parts, which are actually the donor fragments (D) of molecule, and finally 2-(5,6-difluoro-2-methylene-3-oxo-indan-1-ylidene)-malononitrile that act as the peripheral acceptor part (A2) and has direct attachment with the donor fragments making the overall molecule as A2–D–A1–D–A2 type. In this study, modifications were performed at the end-sites of the molecule WR and seven new SFAs (W1–W7) were developed. Thus, the existing terminal acceptor groups, on both sides, were replaced with new electron acceptor groups, *i.e.*, 1-dicyanomethylene-2-methylene-3-oxo-indan-5,6-dicarbonitrile (W1), 6-cyano-1-dicyanomethylene-2-methylene-3-oxo-indan-5-carboxylic acid methyl ester (W2), 1-dicyanomethylene-2-methylene-3-oxo-indan-5,6-dicarboxylic acid dimethyl ester (W3), 2-(6-fluoro-2-methylene-3-oxo-2,3-dihydro-cyclopenta[*b*]naphthalen-1-ylidene)-malononitrile (W4), 2-(1-chloro-5-methylene-6-oxo-5,6-dihydro-cyclopenta[*c*]thiophen-4-ylidene)-malononitrile (W5), 2-(2-bromo-6-methylene-7-oxo-6,7-dihydro-1-thia-*s*-indacen-5-ylidene)-malononitrile (W6), and 2-(2-methylene-3-oxo-2,3-dihydro-cyclopenta[*b*]naphthalen-1-ylidene)-malononitrile (W7). The ChemDraw structures of the above-mentioned WR and W1–W7 molecules are given in Fig. 2.

### 3.2. Method selection and optimized geometries

**Fig. 3:**
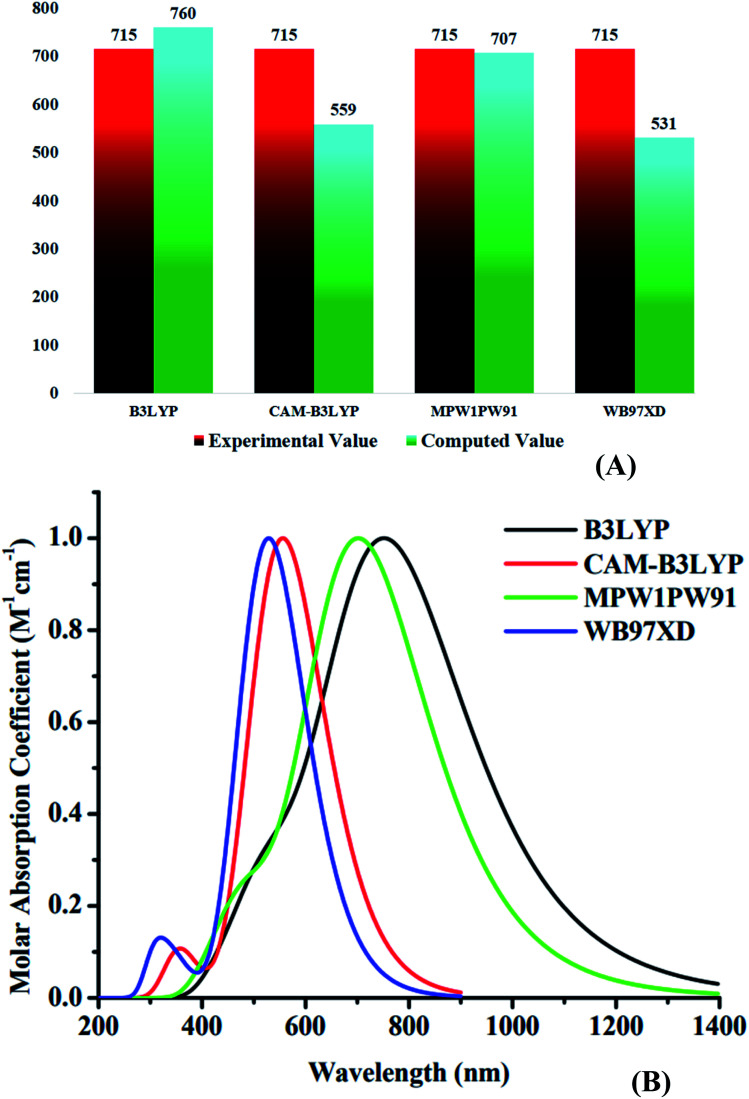
(A) Comparative analysis of computed values with experimental values of WR molecule. (B) Absorption spectra of WR molecule with four different functionals.

Using four different functionals (B3LYP, CAM-B3LYP, MPW1PW91 and ωB97XD) with basis set 6-31G(d,p), the WR molecule was analyzed for its absorptivity measurements. Fig. 3 shows that the *λ*_max_ of WR molecule for these functionals is 760 nm, 559 nm, 707 nm, and 531 nm, sequentially. Experimentally determined *λ*_max_ of WR molecule is ∼715 nm,^32^ and we can see from Fig. 3 that DFT computations based on *λ*_max_ values suggest the MPW1PW91/6-31G(d,p) to have the greatest concordance with the empirically obtained *λ*_max_. So, this functional was chosen to accomplish all auxiliary theoretical calculations of this research. Accordingly, the functional and basis sets were used to optimize all the seven molecules (W1–W7).

**Fig. 4:**
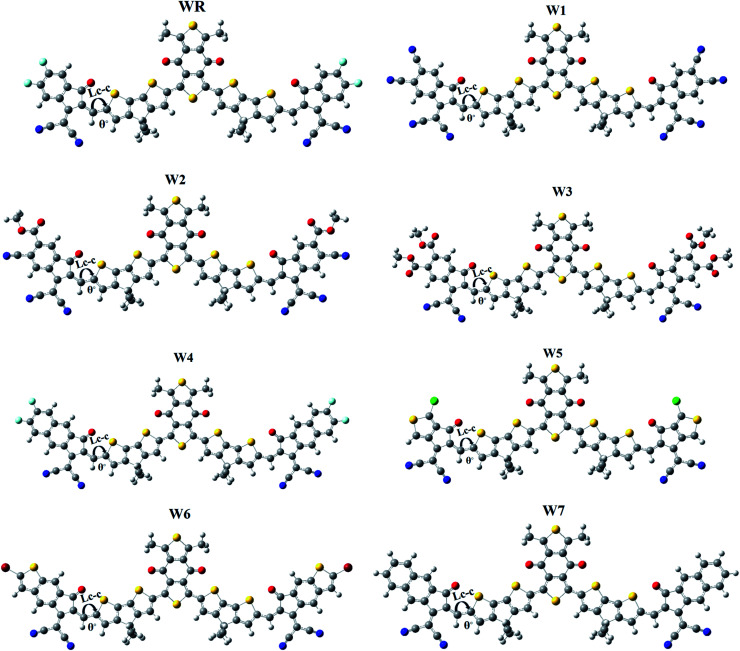
Optimized geometries of WR and W1–W7 molecules.

It is generally known that the optoelectronic characteristics of a molecule are strongly influenced by its molecular structure.^56^ A prolonged conjugation can be observed in the WR and W1–W7 molecules (Fig. 4), resulting from the delocalization of π-electrons, indicating efficient charge transfer in these molecules. The length of bonds (*L*_C–C_), as well as the dihedral angles (θ°), of WR and W1–W7 molecules were calculated (Table 1) to estimate the conjugation and planarity of their molecular structures. It is understandable that the length of single and double bonds between two carbons (C–C and C=C) is 1.54 nm and 1.34 nm, respectively, whereas all compounds analysed had a bond length of 1.40–1.41 nm, at their point of attachment of the A2 acceptors to the donors (pictorially demonstrated in Fig. 4), which implies their increased conjugation and charge transfer capabilities. Furthermore, since the dihedral angles of W1–W7 are so close to that of the significantly planar WR, it can be said that the optimal geometries of all of these compounds have the planar configuration. All of the molecules studied here, exhibited dihedral angles ranging from 0.0008 to 0.2507°, which shows that the newly attached acceptor moieties have not affected the favorable planar topology of the molecules. The greatest dihedral angle amongst all of W3, which is not much as it is, could be attributed to the large ester groups attached at its peripheries.

**Table 1:** Length of bond (Å) and dihedral angles (θ°) of WR and W1–W7 molecules

Molecules	Bond length (*L*_C–C_) (Å)	Bond angle (θ°)
WR	1.41	0.0022
W1	1.40	0.0008
W2	1.40	0.0629
W3	1.41	0.2507
W4	1.41	0.0155
W5	1.41	0.0767
W6	1.41	0.0694
W7	1.41	0.0522

### 3.3. Quantum mechanical descriptors

The highest occupied molecular orbital (HOMO) and lowest unoccupied molecular orbital (LUMO) energies of chromophores have a significant impact on their charge transmission, absorption, as well as electronic properties.^57^ HOMO is the valence band from which electrons are donated, whereas LUMO is in the conductance band in which they are received.^58,59^ Solar cells (SCs) and other photovoltaic (PV) devices are frequently distinguished by their energy gap (*E*_gap_), which actually indicates the necessity of energy for the dissociation of electrons.^60^ The better the efficiency of an organic solar cell, the narrower will be its band gap. A molecule that has the smallest band gap value is critical for constructing PV devices that are both efficient and competent.^61^Table 2 shows the band gaps (*E*_gap_), along with the HOMO and LUMO energies of the WR and W1–W7, as well as some other photophysical characteristics, of the molecules under investigation.

**Table 2:** HOMO, LUMO, *E*_gap_, ionization potential (IP), and electron affinity (EA) of WR and W1–W7 molecules

Molecules	HOMO (eV)	LUMO (eV)	*E*_gap_ (eV)	IP (eV)	EA (eV)
WR	−5.77	−3.51	2.26	6.45	2.84
W1	−6.11	−3.95	2.15	6.74	3.32
W2	−5.91	−3.72	2.18	6.57	3.08
W3	−5.74	−3.51	2.23	6.41	2.86
W4	−5.72	−3.51	2.21	6.37	2.87
W5	−5.75	−3.52	2.22	6.42	2.86
W6	−5.67	−3.42	2.25	6.32	2.79
W7	−5.62	−3.39	2.23	6.27	2.76

**Fig. 5:**
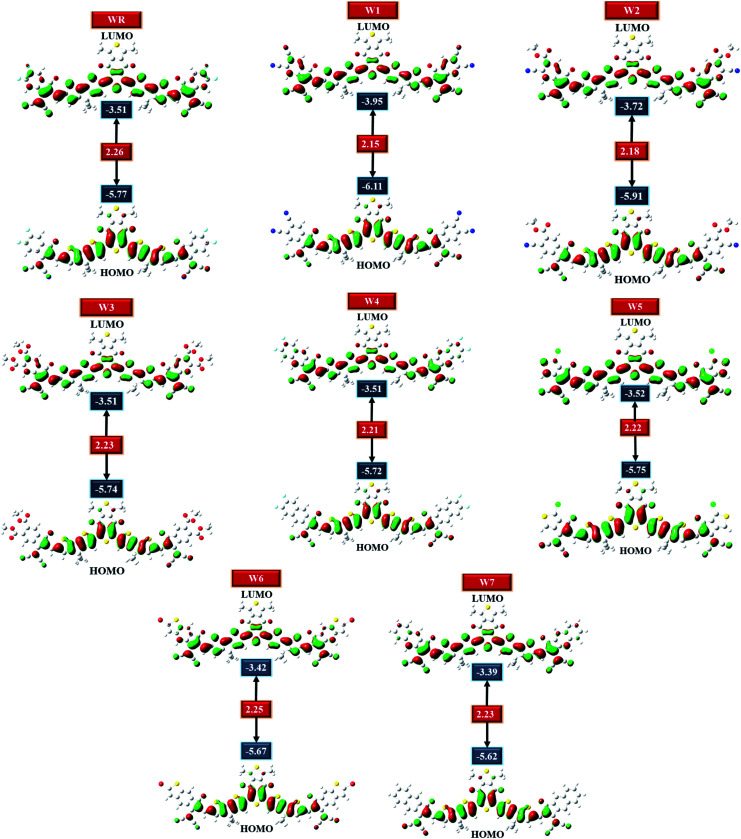
HOMO and LUMO of WR and W1–W7 molecules.

HOMO–LUMO plots of all molecules (constructed from their optimized geometries) under investigation with their respective *E*_gap_ are shown in Fig. 5. Charge density in the ground state (HOMO) is concentrated on the molecule's core acceptors (A1) and donors (D), but this charge density seemed to also move towards the molecule's end-group acceptors when excited (LUMO), clearly demonstrating the efficiency of transfer of charge from the ground states to the excited states. Moreover, the spread of charge density over almost the whole molecule in both the evaluated FMOs, signifies the molecule's prominently planar topology.

HOMO and LUMO energies for the WR molecule have been measured at −5.77 eV and −3.51 eV, respectively, and this molecule showed the bandgap (*E*_gap_) of 2.26 eV, which is greater than all our newly created molecules. W1 molecule demonstrated the lowest HOMO energy amongst all, which means that the HOMO of W1 is the most stable of all the molecules studied. End-capped acceptor groups of W1 molecules include pairs of strongly electron withdrawing cyano groups, which could contribute to this stability. Additionally, the W2 molecule has the second most stable HOMO after W1, because of its lower HOMO energy level of −5.91 eV, and just like W1, this molecule also has a greater number of cyano groups than all other molecules. This could mean that the cyano groups played a contributing role in the stability of the W1 and W2 molecules. Overall, the decreasing arrangement for HOMO energy level of all the studied molecules is W7 > W6 > W4 > W3 > W5 > WR > W2 > W1, and the decreasing sequence of LUMO energy level for WR and W1–W7 molecules is W7 > W6 > W4 = W3 = WR > W5 > W2 > W1. So, in both the evaluated FMOs, W1 and W2 have the lowest and second lowest energy levels, respectively. Moving on, W1 molecule also has a smaller *E*_gap_ (2.15 eV) in contrast to WR and W2–W7 molecule. And once again, W2 molecule exhibited the second smallest *E*_gap_ (2.18 eV). Furthermore, the *E*_gap_ of all the newly constructed molecules are smaller than the WR molecules, which portrays the point that the modified molecules have quite a worth of efficient charge transfer capabilities. The declining sequence of all studied molecule for the *E*_gap_, as evaluated from Table 2, is WR > W6 > W7 = W3 > W5 > W4 > W2 > W1.

### 3.4. Ionization potential and electron affinity

Studies of charge transfer efficiency can be directed by factors, such as ionization potential (IP) and electron affinity (EA). Here, IP is the amount of energy required by a molecule for the donation of its electrons, while EA is that energy, which is released over the addition of electron to a molecule.^62^ Molecules with small IP and EA values exhibit ability to donate electrical charge efficiently, reason is that the electron donor moieties disrupt the HOMO energy state by facilitating electron transportation, hence causing a smaller IP and EA, while molecules with strong electron-pulling groups possess higher IPs and EAs, because of the stability of the HOMO as a result of which electron removal becomes more difficult.^63^ In present research, IP and EA values have been estimated using eqn (3) and (4).^64^

3 IP = [*E*_0_^+^ − *E*_0_]

4 EA = [*E*_0_ − *E*_0_^−^]

The estimated values of IP and EA for WR and W1–W7 molecules are listed in Table 2. W7 molecule has the smallest IP (6.27 eV) amongst all molecules, the reason being its destabilized HOMO energy level (−6.62 eV). W1 has the largest EA (3.32 eV) because of its stabilized HOMO level (−6.11 eV). Second largest EA (3.08 eV) value, when compared to all other considered molecule, is shown by W2 molecule. This is because of most stabilized HOMO (−5.91 eV) energy level of W2. Overall, the greater IP and EA of W1 and W2 molecules, illustrates their efficient abilities as acceptor components in the organic PV cells. Also, the greater EA values of W3–W5 molecules, favors the better abilities of these molecules as acceptors in comparison to WR.

### 3.5. Absorption spectrum

**Fig. 6:**
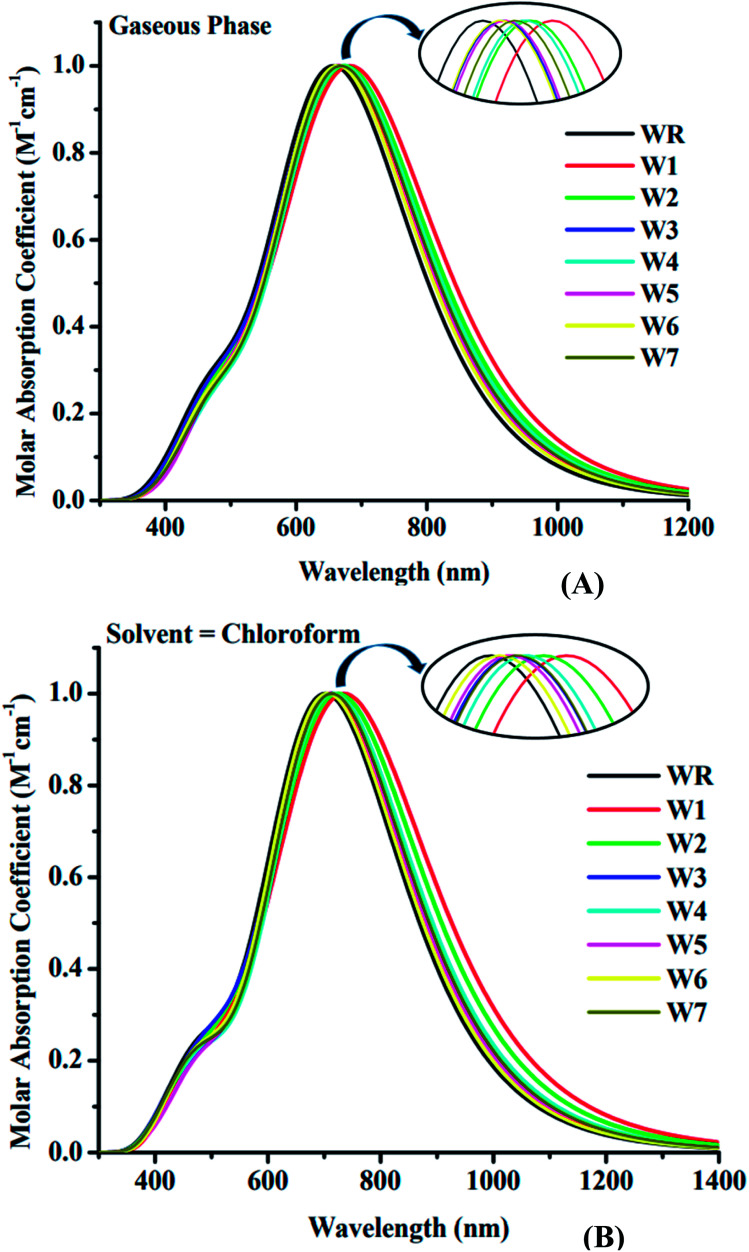
Absorption spectra of WR and W1–W7 molecules in (A) gaseous phase and (B) CHCl_3_ solvent.

The spectral analysis of WR and W1–W7 molecules was done by employing the above determined functional and basis set, *i.e.*, MPW1PW91/6-31G(d,p), and the estimated data in both the gaseous and the solvent (chloroform) phase is given in Tables 3 and 4. All of the compounds under investigation have an absorbance spectrum ranging from 350 nm to 1400 nm in both the studied phases, as shown in the Fig. 6. In gaseous phase, the WR molecule's *λ*_max_ is 663 nm, whereas in chloroform (CHCl_3_), it is 707 nm. The *λ*_max_ of Y6 acceptor molecule is 731 nm in CHCl_3_ solvent.^14^ On the other hand, W1–W7 molecules have the *λ*_max_ range from 668 nm to 691 nm and 711 nm to 748 nm in the gaseous phase and CHCl_3_ solvent, respectively. This UV-visible spectral analysis of all the considered molecules reveals that all of the altered molecules had a bathochromic-shift in their *λ*_max_ in both phases (gaseous and solvent) when compared to the WR molecule, and this bathochromic shift shown by W1–W7 molecules is within the range of 5 nm to 28 nm and 8 nm to 41 nm, with respect to WR molecule in gas and solvent phase, respectively. Inclusively, the increasing *λ*_max_ sequence of WR and W1–W7 molecules in gas phase and in the CHCl_3_ is WR < W6 < W3 < W5 < W7 < W4 < W2 < W1 and WR < W6 < W5 < W7 < W3 < W4 < W2 < W1, sequentially. Because of the reason that the polar excited state was stabilized with the assistance of a polar solvent,^65^ all of the compounds demonstrated a bathochromic shift in chloroform solvent as opposed to when in gas phase. Because of the inverse relationship between energy and wavelength, the reduced *E*_gap_ of all the molecules caused their wavelength to increase.^66^ When compared to the absorption maxima of the Y6 molecule, both the W1 and W2 molecules have shown a red-shift in their respective *λ*_max_ values. W1 and W2 molecules exhibit a 17 nm and a 5 nm bathochromic shift, respectively, when compared to Y6. Findings show that W1 has the greatest *λ*_max_ in both phases, specifically, 691 nm in gas and 748 nm in solvent medium, indicating the existence of prominent end-capped acceptor (1-dicyanomethylene-2-methylene-3-oxo-indan-5,6-dicarbonitrile) that might have influenced its wavelength of maximum absorption (*λ*_max_).

**Table 3:** The *λ*_max_, excitation energy (*E*_x_), oscillator strength (*f*) and assignment of WR and W1–W7 molecules in gaseous phase

Molecules	Calculated *λ*_max_ (nm)	*E*_x_ (eV)	(*f*)	Assignment
WR	663	1.87	2.88	H → L (+96%)
W1	691	1.79	2.93	H → L (+96%)
W2	682	1.82	2.96	H → L (+96%)
W3	670	1.85	3.01	H → L (+96%)
W4	677	1.83	3.16	H → L (+96%)
W5	671	1.85	2.93	H → L (+97%)
W6	668	1.86	3.22	H → L (+96%)
W7	674	1.84	3.15	H → L (+96%)

**Table 4:** The *λ*_max_, excitation energy (*E*_x_), oscillator strength (*f*) and assignment of WR and W1–W7 molecules and *λ*_max_ and *E*_x_ of Y6 molecule in CHCl_3_ solvent

Molecules	Exp. *λ*_max_ (nm)	Calculated *λ*_max_ (nm)	*E*_x_ (eV)	(*f*)	Assignment
WR	∼715	707	1.75	3.18	H → L (+95%)
W1	—	748	1.66	3.08	H → L (+94%)
W2	—	735	1.69	3.17	H → L (+94%)
W3	—	720	1.72	3.21	H → L (+94%)
W4	—	722	1.72	3.46	H → L (+95%)
W5	—	715	1.73	3.28	H → L (+95%)
W6	—	711	1.74	3.49	H → L (+94%)
W7	—	718	1.73	3.46	H → L (+95%)
Y6	731	—	1.33	—	—

**Fig. 7:**
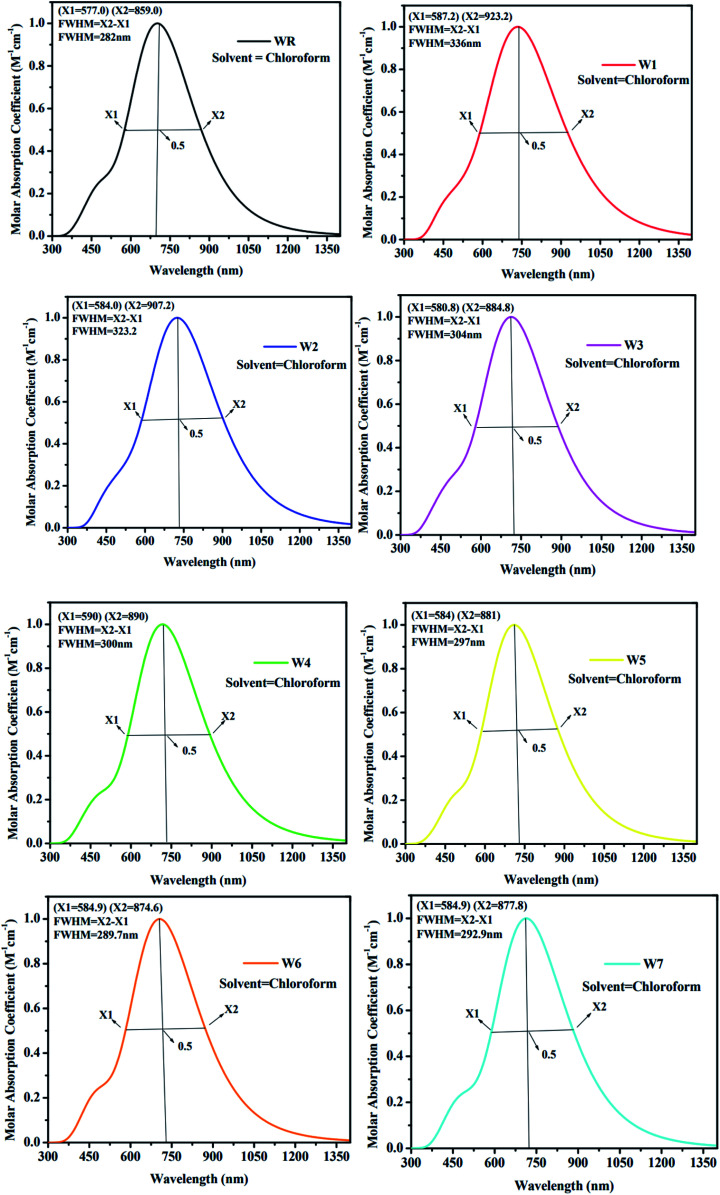
FWHM estimations of WR and W1–W7 molecules.

As an additional measure, in chloroform solvent, the full width half maximum (FWHM) of all new molecules (W1–W7) was computed other than the measurement of *λ*_max_ and was then compared to the FWHM of WR molecule.^67^ The graphical representation of FWHM of WR and W1–W7 molecules is shown in Fig. 7. The FWHM of WR is 282 nm and of W1–W7 molecules showed the values of 336 nm, 323.2 nm, 304 nm, 300 nm, 297 nm, 289.7 nm, and 292.9 nm, respectively. The FWHM of all freshly formulated molecules is greater than that of WR molecules, which clearly demonstrates that new molecules have better absorption profile than their reference molecule. W1 molecule with the greatest *λ*_max_, also have greatest FWHM, and according to the study's findings, its absorption profile is superior to that of any other studied molecule in this research work. Overall, it can be said that all our designed acceptor molecules would be efficient candidates for solution manufacturing of organic solar cells.

A dimensionless factor called oscillator strength (*f*), is critical for the purpose of determining the optical aspects of photovoltaic cells and for computing the generated intensity of radiation when there is electrical excitation between two energy levels.^68^ Excitation energy (*E*_x_) is the energy required for the probable transition; consequently, increasing the *f*, lowering the *E*_x_, and widening the absorption spectra, all project towards an effective ICT. The W1–W7 molecules have larger *f* and smaller *E*_x_ than the WR molecule, hence they could possess superior ICT. Y6 has smallest *E*_x_ (1.33 eV)^69^ than all other studied W-shaped (WR, W1–W7) acceptor molecules (Tables 3 and 4).

### 3.6. Light harvesting efficiency (LHE)

Every material utilized in the SCs must have the capacity to create charge after light collecting which is quite often called as LHE.^70^ The oscillator strength has a significant impact on LHE, which in turn has a substantial impact on the short-circuit current production, and thus this LHE influences the device's solar efficiency. All of the compounds investigated had their LHE calculated by using eqn (5).^60^

5 *η*_λ_ = 1 − 10^−*f*^

where *f* stands for oscillator strength (values taken from Table 4) and *η*_λ_ is the LHE. Table 5 lists the LHE values computed of WR and W1–W7 molecules in the chloroform solvent. As a result of their greater oscillator strengths, W3–W7 molecules showed higher LHE values than WR. LHE is highest in W6 because of its stronger oscillation strength. According to the results, the terminal acceptor moieties have a significant impact on the LHE of these small molecules.

**Table 5:** LHE of WR and W1–W7 molecules

Molecules	LHE
WR	0.99933
W1	0.99916
W2	0.99932
W3	0.99938
W4	0.99965
W5	0.99947
W6	0.99967
W7	0.99965

### 3.7. Dipole moment

Crystallinity, as well as solubility, are two of the most important factors that are majorly influenced by dipole moment (*D*). These factors are critical in determining polarization phenomena in the needed solvent for effective organic solar systems.^71^ Planar and organized geometries of molecules with significant dipole moments allows continuous charge transfer because of their tight molecular assembling, better crystallinity, and greater solubility in polar solvents. The more dipole moment a molecule has, the greater its crystallinity and solubility in polar substances is, both of which contribute to enhanced charge transmission.^72^ Organic polar solvents, such as chloroform, cannot generally dissolve molecules with zero dipole moment. Nevertheless, this relationship is not universal, since each molecule has its own unique molecular arrangement that impacts its solubility and charge transfer capabilities. Table 6 shows the *D* values of molecules WR and W1–W7 in both gaseous and solvent states, and in both states, all the molecules follow the same increasing sequence, *i.e.*, W1 < W5 < WR < W2 < W4 < W6 < W3 < W7. The *D* of the W1 and W5 molecules was lower than the WR molecule, but that of the other molecules was higher than our reference molecule. With regards to its polarity and solubility, W7 had the biggest dipole moment value, which may be explained by its dibenzene rings containing cyano groups at its terminal ends.

**Table 6:** Dipole moment (*D*) of WR and W1–W7 molecules in gas phase and CHCl_3_ solvent

Molecules	(*D*) (gaseous phase)	(*D*) (CHCl_3_ solvent)
WR	5.227	5.687
W1	4.118	5.387
W2	5.741	5.924
W3	7.698	8.229
W4	6.389	7.281
W5	5.135	5.433
W6	6.585	7.368
W7	9.152	10.483

### 3.8. Density of states (DOS)

**Fig. 8:**
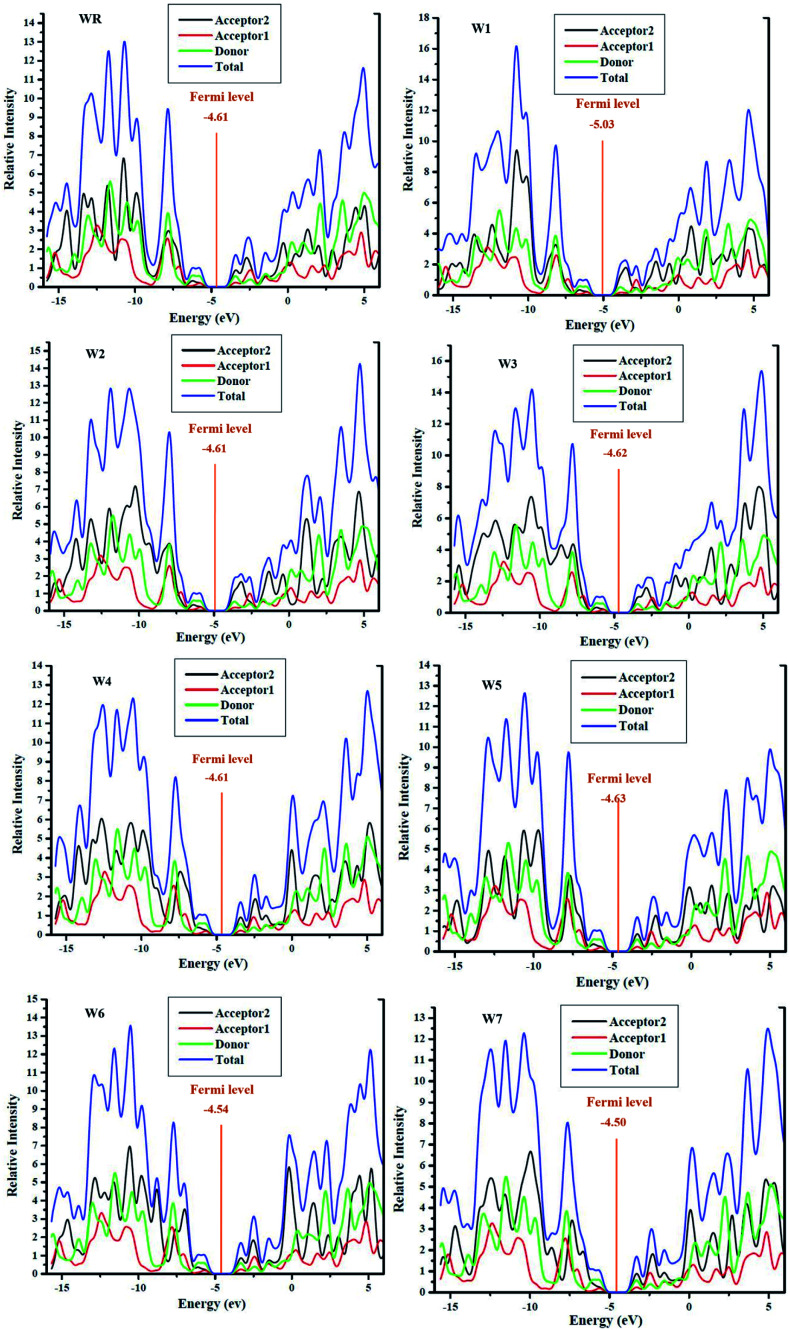
DOS plots with mentioned Fermi level of WR and W1–W7 molecules.

It is important to conduct DOS studies, in order to better understand how the functions of each molecules' fragments (donor, acceptor1 and acceptor2) and overall performances of molecules are validated in terms of their respective DOS, *i.e.*, partial and total DOS, respectively, in the molecules' charge mobility.^73^ It is an important factor in determining how frontier molecular orbitals (FMOs) are arranged in terms of the Mulliken charge density. Using the MPW1PW91/6-31G(d,p) methodology, DOS calculations of all the molecules investigated were performed, and the plots were created using PyMOlyze 1.1 software. There is an *x*-axis for the energy and a *y*-axis for the corresponding relative intensity, in the DOS plots. In the lots, the peaks to the left of the central planar zone (the bandgap) illustrates the HOMO levels, while those towards its right signifies the LUMO energy levels. In this study, each molecule was fragmented into three portions, namely, donor, acceptor1, and acceptor2, for the investigation of the contributions of each fragments to the FMOs. In Fig. 8, the contributions of the acceptor2, acceptor1, donor moieties, as well as the total contribution of the molecules (WR and W1–W7) as a whole, in raising the FMOs can be seen as black, red, green, and blue lines, respectively. Moreover, the numerical contribution of individual segment of WR and W1–W7 molecules is listed in Table 7.

**Table 7:** Involvement of donor, acceptor2, and acceptor1 fragments in the formation of FMOs of WR and W1–W7 molecules

Molecules		Donor (%)	Acceptor2 (%)	Acceptor1 (%)
R	HOMO	57.8	21.3	21.0
	LUMO	38.2	47.7	14.0
W1	HOMO	56.1	22.8	21.1
	LUMO	34.2	55.2	10.6
W2	HOMO	56.7	22.5	20.8
	LUMO	34.9	53.6	11.4
W3	HOMO	57.3	22.0	20.7
	LUMO	36.7	50.3	13.0
W4	HOMO	56.8	22.9	20.3
	LUMO	36.9	49.8	13.3
W5	HOMO	56.8	22.6	20.5
	LUMO	38.3	47.9	13.7
W6	HOMO	57.4	22.3	20.4
	LUMO	38.4	47.3	14.3
W7	HOMO	57.0	22.8	20.2
	LUMO	37.3	48.8	14.0

For all the newly developed and the WR molecule, the donor group is the largest contributor to the elevation of ground state (HOMO). The acceptor1 and acceptor2, both have comparable but low contribution in HOMO energy level, although acceptor2 showed a bit more contribution than acceptor1. The major contributor of the excited state (LUMO) is acceptor2. These results evaluate that charges are to be transported from the electron rich fragments (donor) of molecules at the ground state to the electron withdrawing fragments (acceptor2) of molecules in the excited state *via* successive conjugation. In addition, the FMO analysis of the reference and developed compounds is likewise supported by these findings, as shown in Fig. 5.

Fermi levels were used to estimate the chance that an electron would be found in either the HOMO or LUMO. Having a Fermi level near to the LUMO indicates that electrons may easily go from the ground state to the excited state, or that the electrons reside in the excited state more often. Fermi level of WR and W1–W7 molecules are shown in their DOS graphs represented in Fig. 8.

### 3.9. Electrostatic potential (ESP)

**Fig. 9:**
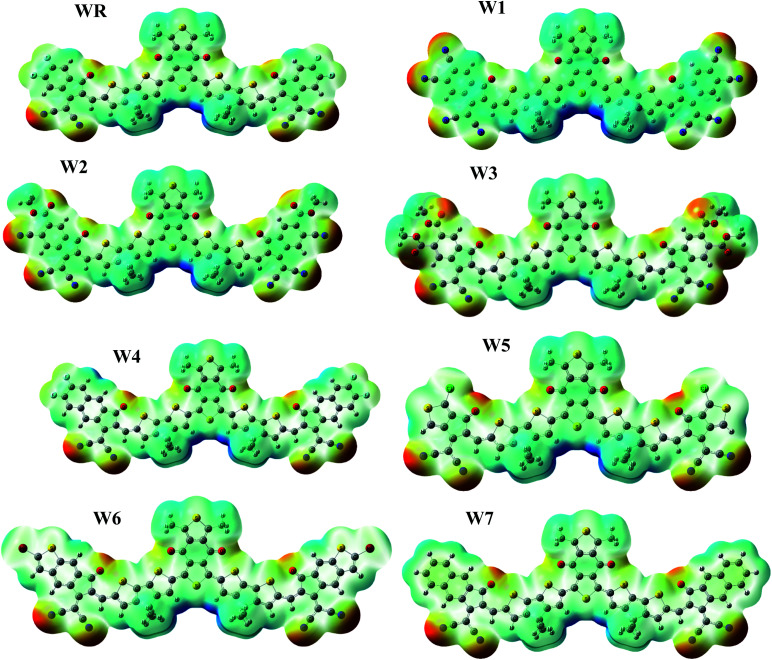
ESP maps of WR and W1–W7 molecules.

ESP depicts the three-dimensional (3-D) charge dispersion that occurs in a molecule, as well as highlights the molecule's different sites with respect to the existence of electrons.^74^ For the purpose of predicting the reactivity of a molecular frameworks, ESP analysis was performed on our scrutinized molecules. ESP maps are the 3-D arrangement of electrons, lone pairs, and electronegative substances that are easily accessible to nucleophilic action. On the ESP maps, red denotes a negative zone with high electron existence, green denotes neutral spots, and blue denotes a positive area with low electron concentration. The ESP colored maps of WR and W1–W7 are shown in Fig. 9.

On ESP maps, the nitrogen and oxygen atoms at the peripheral acceptor regions of the molecules appear dark red, indicating the presence of prominent electron density over these sites. The oxygen atoms of the acceptor part of the core (A1) also depict red on the maps, hence the presence of electron density across these spots is readily visible. These ESP maps show that the donor parts containing thiophene rings, as well as methyl groups, have a severe lack of electrons on certain places of the molecules, which is shown by a blue color.

### 3.10. Analysis of charge mobility

**Fig. 10:**
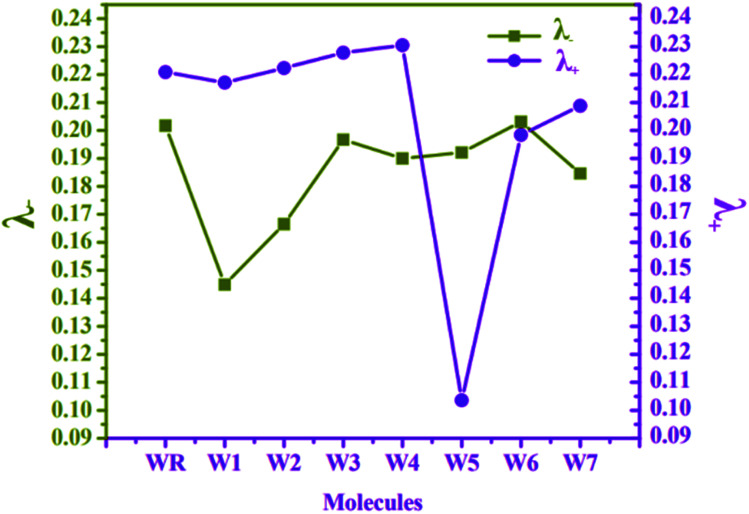
RE plot of *λ*_−_ and *λ*_+_ for WR and W1–W7 molecules.

At MPW1PW91 functional, reorganization energies (RE) of W1–W7 and WR molecules were calculated to investigate the charge transfer mobility from electron donor to acceptor fragments. It is the RE, which measures the amount of charge that could be transferred from donor to acceptor components of a molecule, and is associated with the mobility of hole and electron charge, which is the primary driving force behind the development of proficient materials for OSCs.^75^ The mobility of these charges (electrons and holes) is actually inversely proportional to the RE.^76^ Because of this, the charge transfer will be more efficient if the value of the RE is lowered. Cations and anions' geometrical arrangements are among the numerous variables that influence the RE. Both the anionic and cationic geometries point to the movement of electrons and holes in between the donor and the acceptor units. In accordance with eqn (1) and (2), the RE values of electrons and holes for all of the studied molecules were determined, and the results are listed and presented in Table 8 and Fig. 10, respectively.

**Table 8:** RE of *λ*_−_ and *λ*_+_ for WR and W1–W7 molecule

Molecules	λ_− (electron)_	*λ*_+ (hole)_
WR	0.2017	0.2209
W1	0.1449	0.2171
W2	0.1665	0.2223
W3	0.1967	0.2278
W4	0.1900	0.2305
W5	0.1921	0.1036
W6	0.2031	0.1984
W7	0.1846	0.2088

The RE of WR molecule for *λ*_−_ and *λ*_+_ is 0.2017 eV and 0.2209 eV, respectively. All the altered molecules, except W6, have lower RE energies for electron than the reference molecule, making them better than WR molecule in terms of their electron mobility. In comparison to the other molecules tested, the RE of molecule W1 was found to be the lowest (0.1449 eV), indicating that the new terminal acceptors in this molecule have played an important role in boosting its electron mobility. The sequence of molecule with respect to their *λ*_−_ is W1 < W2 < W7 < W4 < W5 < W3 < WR < W6. It can be said that till now W1 molecule has proved itself to be the best acceptor molecule amongst all.

The *λ*_+_ of W1 and W5–W7 molecules is less than that of WR, which means that they have superior hole mobility than WR. The W5 molecule's hole RE has decreased dramatically, showing that the end-group acceptor is actively contributing to the reduction of the molecule's RE in order to its improve hole mobility. Concisely, W5 < W6 < W7 < W1 < WR < W2 < W3 < W4 is the order of all the molecules for hole RE. The RE of a very widely known acceptor molecule Y6 for *λ*_−_ and *λ*_+_ is 0.15 eV and 0.16 eV^77^ and by comparing these energies with our newly proposed molecules, it was estimated that W1 and W5 molecules are better than Y6 molecule in the aspect of electron and hole mobilities, respectively. Overall, the low *λ*_−_ values of all molecules in comparison to their *λ*_+_ values, except for W5, signifies their enhanced abilities to act as acceptors in advanced organic photovoltaic cells.

### 3.11. Transition density matrix (TDM) and binding energy (*E*_b_)

**Fig. 11:**
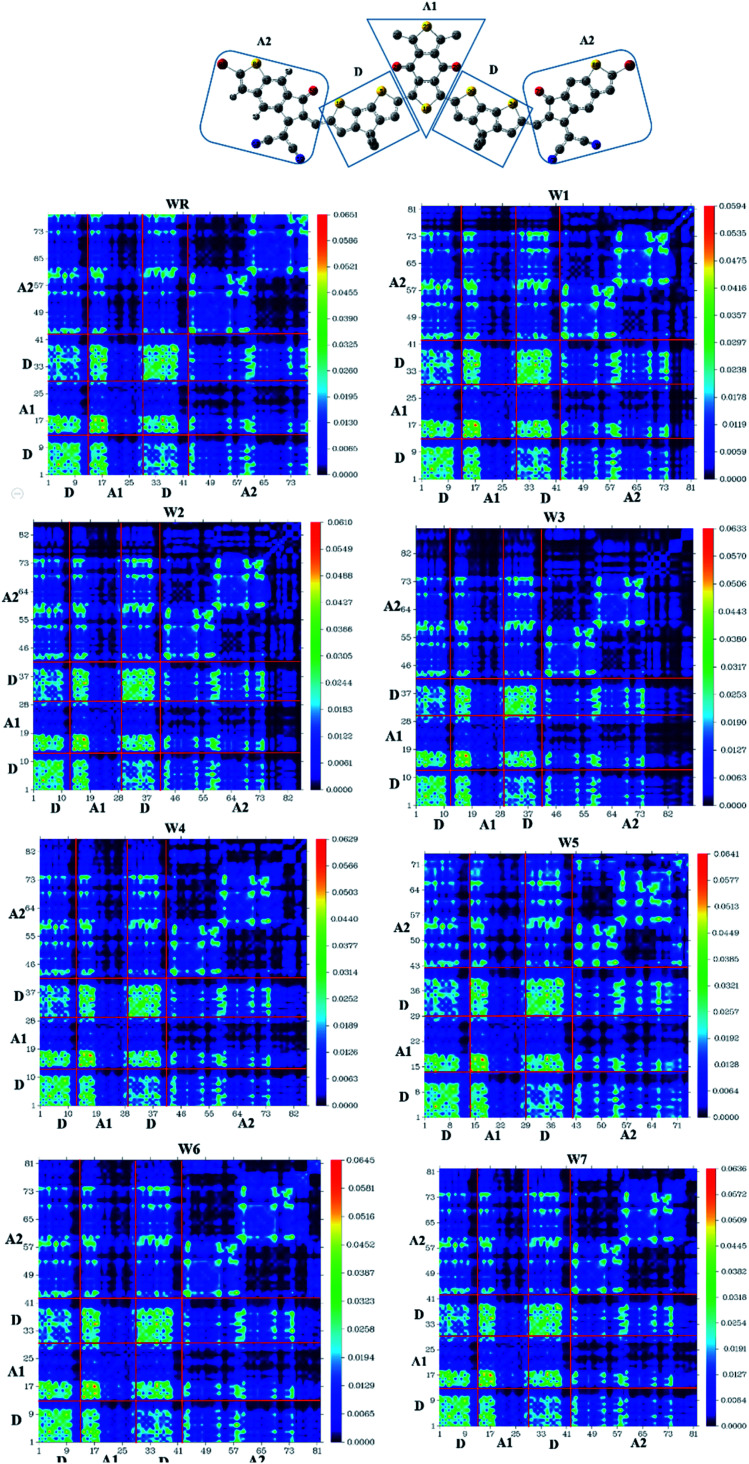
TDM plot of WR and W1–W7 molecules.

TDM analysis seems to be necessary in prediction of the exciton (electron hole pair) transition between donor–acceptor areas at particular locations in conjugated chemical systems.^78,79^ Predicted charge transitions, fundamental charge locations, and imaging of exciton mobility during emission, as well as absorption processes, in an excited state could all be examined in this analysis.^80^ In order to understand the mobility of charges within a molecule, and to evaluate electronic properties, such as effects of resonance, as well as extent of delocalization, a TDM plot is commonly used. Hydrogen's role in the transition is mostly ignored in TDM analysis because of its insignificance in charge transfer-abilities. Matrix numbering on bottom *x*- and left *y*-axes represent all the atoms, except hydrogen, while colored bars starting with blue color and ending up on red, on the right *y*-axis, indicate charge density coefficient. It was necessary to divide the molecule into three parts in order to calculate the transition pathway, and these three parts were designated as donor (D), acceptor1 (A1), and acceptor2 (A2), as shown in Fig. 11.

It is observed for all of the compounds that the charge density is dispersed effectively throughout the molecule, in both diagonal and off-diagonal patterns, with the diagonal pattern accounting for the majority of the charge dispersion. The flow of charge could be identified to be moving effectively from donor to acceptor1 and acceptor2, suggesting that the entire molecule is undergoing sequential conjugation.

Interaction Coefficient of WR and W1–W7 molecules, in the chloroform solvent, is given in Table 9 and follows the increasing order of W1 < W2 < W3 < W6 < W7 < W4 < WR < W5. A low interaction coefficient of W1 indicates that the donor and acceptor parts of this molecule are able to efficiently transmit electrons from one to the other. This is because of the reason that a low interaction coefficient points towards the greater mobility of charges in the molecule.^53^

**Table 9:** *E*_gap_, *E*_b_ (gaseous), *E*_b_ (chloroform solvent), and interaction coefficient of WR and W1–W7 molecules and *E*_gap_ and *E*_b_ of Y6 molecule

Molecules	*E*_gap_ (eV)	*E*_b_ (eV) gaseous	*E*_b_ (eV) solvent	Interaction coefficient
WR	2.26	0.39	0.51	0.68850
W1	2.15	0.36	0.49	0.68584
W2	2.18	0.36	0.49	0.68639
W3	2.23	0.38	0.51	0.68713
W4	2.21	0.38	0.49	0.68780
W5	2.22	0.37	0.49	0.68975
W6	2.25	0.39	0.51	0.68736
W7	2.23	0.39	0.50	0.68766
Y6	1.55	—	0.22	—

Another factor to consider here, is the binding energy of the excitons. Organic solar cells may be tested for their electron–hole (exciton) dissociation possibilities, operating efficiency, and electrical properties using the binding energy.^81^*E*_b_ is the determination of the interactions between the electron and the hole's coulombic forces.^82^ Low *E*_b_ indicates that there is less coulombic interaction between electrons and holes, and vise-versa. Eqn (6)^83^ was used to calculate *E*_b_ values in presented in Table 8.

6 *E*_b_ = *E*_gap_ − *E*_x_

*E*_gap_ above is the HOMO–LUMO gap and *E*_x_ is the first excitation energy, either in the gas or solvent phase. Sun *et al.* computed the *E*_b_ of six polymers produced from polythiophene by the use of range separated functional with polarizable continuum model.^83^ In the gaseous phase, W1–W5 molecules have lesser *E*_b_ values than WR molecules, while W1, W2, W4, W5, and W7 molecules have smaller *E*_b_ values in the chloroform solvent, with respect to WR. It should be noted that the *E*_b_ of the remaining two molecules, W3 and W6, is also comparable to the reference molecule. *E*_b_ of Y6 is 0.22 eV^69^ and from the results it is estimated that WR, W1–W7 molecules have larger *E*_b_ than Y6 molecule. All the molecules have a little higher *E*_b_ in chloroform solvent as compared to in the gaseous form, the reason is that the polar solvent must have interacted and bonded directly with excitons. Excitons of molecules that have smaller *E*_b_ values readily diffuse into free charge carriers, making them an appropriate choice for increased current charge density, as the faster these charge carriers (electrons and holes) move towards their respective electrodes, the greater will be the current produced, hence greater will the efficiency of the device. So, the lowest binding energy amongst all, seems to be that of the W1 and W2 molecules in both the evaluated phases, making the most proficient candidates amongst all.

### 3.12. Device performance

**Fig. 12:**
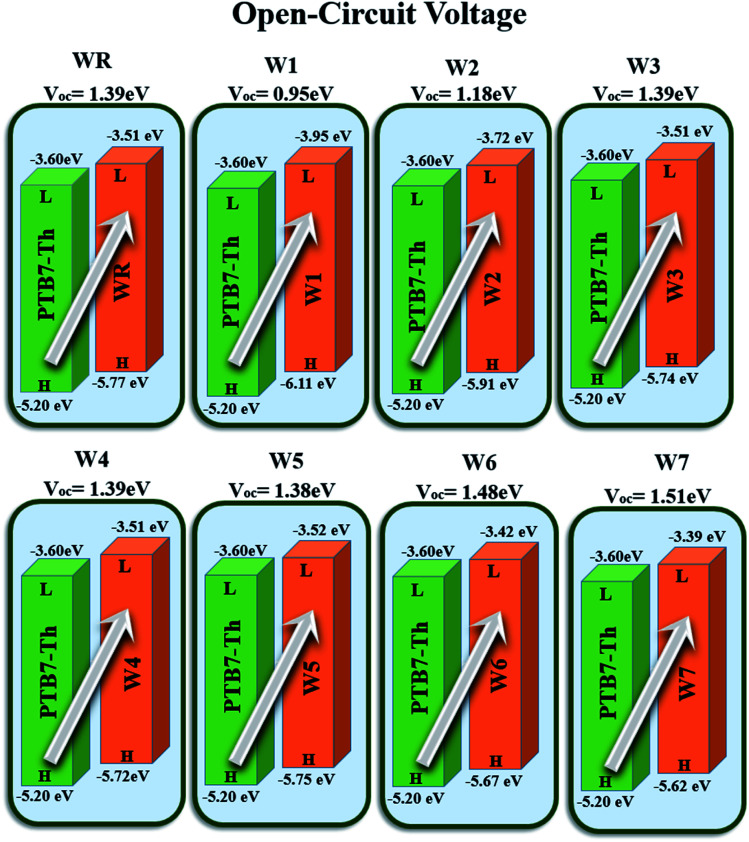
Theoretically predicted *V*_OC_ of WR and W1–W7 acceptor molecules by pairing them with PTB7-Th donor.

Open circuit voltage (*V*_OC_) can be used to evaluate the photovoltaic activity of any solar instrument, and is an important part in figuring out how the instrument works.^84^*V*_OC_ truly represents the entire possible voltage provided by an optical device, when no external load is present.^85^ Various photovoltaic features influence the *V*_OC_, such as incident light, charge transfer, temperature of the solar device, *etc*.^86^ The donor material's valence band is usually coupled with the acceptor material's conduction band, resulting in optimum voltage. A lower HOMO level in the donor compound and a greater LUMO level in the acceptor compound are required for the increased *V*_OC_ values. An increase in *V*_OC_ results in an increase in fill factor, which is the basis for a photovoltaic system's high PCE.^87^ When it comes to determining the performance of a solar device, *V*_OC_ and intermolecular energy gaps are directly linked. To achieve the highest possible *V*_OC_, the HOMO level of polymer donor PTB7-Th was paired with the LUMO of the reference and newly assumed small acceptor molecules in the current study (WR, W1–W7). According to the available research, PTB7-Th is a reliable donor with HOMO energy of −5.20 eV and LUMO of −3.60 eV.^88^ In this research, *V*_OC_ was estimated statistically using the following eqn (7):^70^7
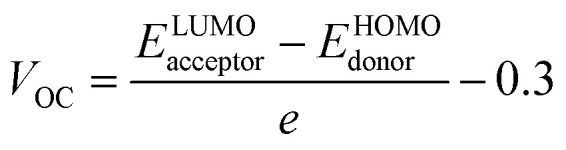
*e* represents the molecular charge that is 1 in the equation above, while inter-surface charge is another factor with general used value of 0.3. The *V*_OC_ of WR was found to be 1.39 eV and the range of *V*_OC_ for W1–W7 molecules was; 0.95–1.51 eV. When compared to WR, the *V*_OC_ of W3 and W4 molecules is almost same. W6 and W7 molecules, on the other hand, have a higher *V*_OC_ than the WR molecule. The Fig. 12 depicts theoretically calculated *V*_OC_ levels for all studied compounds in relation to PTB7-Th, whereas Table 10 provides the statistically calculated data. WR and W1–W7 molecules followed the increasing order of W1 < W2 < W5 < WR = W3 = W4 < W6 < W7. The W7 molecule has the highest *V*_OC_ value according to this investigation, hence it may be used to improve the PCE of OSCs.

**Table 10:** *V*_OC_, normalized *V*_OC_ and FF of WR and W1–W7 molecules

Molecules	*V*_OC_ (eV)	Normalized *V*_OC_	FF
WR	1.39	53.76	0.9087
W1	0.95	36.74	0.8775
W2	1.18	45.64	0.8963
W3	1.39	53.76	0.9087
W4	1.39	53.76	0.9087
W5	1.38	53.38	0.9082
W6	1.48	57.24	0.9131
W7	1.51	58.41	0.9144

The fill factor (FF) of PV systems is one of the most important parameters in determining their PCE, since both are directly related to one another. The *V*_OC_ at the acceptor and donor molecules' interface has a significant impact on this aspect. Eqn (8),^89^ shown below, was used to compute the FF of all our studied molecules.8
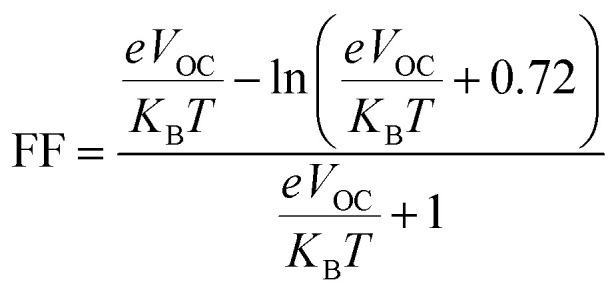

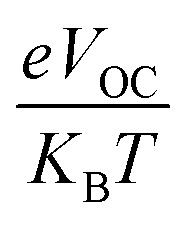
 is the normalized *V*_OC_, in which the standard charge (*e*) is always 1, *K*_B_ denotes the Boltzmann constant (8.61 733 034 × 10^−5^ electron volts per Kelvin) and a constant temperature (300 K) is denoted by the letter *T*. Table 10 lists the normalized *V*_OC_ and FF, along with the *V*_OC_ values for the compounds WR and W1–W7 that were calculated using computational approaches. The FF and normalized *V*_OC_ of WR molecule is 0.9087 and 53.76, respectively. Because of the greater *V*_OC_ of the W6 and W7 molecules, their normalized *V*_OC_ (57.25 and 58.4) and FF (0.9131 and 0.9144) are higher than those of the WR molecule, hence the studies show that the exceptional photoelectronic properties of these small molecules might make them effective for practical use.

Consolidating all solar cell performance parameters into one statistic, *i.e.*, the power conversion efficiency (PCE), which is used to ensure that the photovoltaic material is efficient enough for use in practical applications.^90^ The *V*_OC_, the FF, and the short circuit voltage (*J*_SC_) all have a direct effect on the PCE of a molecule, while the power of radiation that hits the cell interface has an opposite effect. Eqn (9)^12^ explains this relationship quite effectively.9
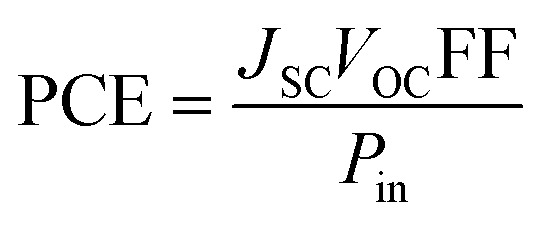
Some of the factors present in preceding equation, *i.e.*, *V*_OC_, and FF, have been theoretically estimated for WR and W1–W7 molecules in this work, and though the *J*_SC_ has not been calculated due to the limited resources attributed to this being a theoretical work, one of the factors of the *J*_SC_ has been evaluated above, specifically, the LHE. All the results show that the PCE of W3 and W4 molecules would be almost equivalent to that of WR, but the *V*_OC_, FF, and LHE of W6 and W7 molecules are better than WR, suggesting that they may have a higher PCE than the WR molecule.

## 4 Conclusion

In the current study, with the objective to boost the efficiency of organic solar cells, seven new W-shaped small molecule acceptors (W1–W7) of A2-D-A1-D-A2 were developed theoretically by the end-group alteration of the reference (WR) molecule. The MPW1PW91 functional with the 6-31G(d,p) basis set was used to explore the various optoelectronic properties of the WR and W1–W7 molecules, *i.e.*, their HOMO–LUMO energy levels, *E*_gap_, ionization potential, electron affinity, absorption maxima (*λ*_max_), full width half maxima, oscillator strength, dipole moment, excitation energy, density of states, light harvesting efficiency (LHE), transition density matrix, and open circuit voltage (*V*_OC_), *etc.* The results revealed a bathochromic shift in the absorption maxima (*λ*_max_), reduced HOMO–LUMO gap (*E*_gap_), and smaller excitation energy (*E*_x_) of the altered molecules as compared to the WR molecule. The range of *λ*_max_, *E*_gap_, and *E*_x_ (in solvent phase) is 711–748 nm, 2.25–2.15 eV and 1.66–1.74 eV, respectively for all the newly modified molecules. Among all the altered molecules, W1 showed the highest *λ*_max_ (748 nm in chloroform), the smallest *E*_gap_ (2.15 eV), and smallest *E*_x_ (1.66 eV in the solvent phase). When it came to LHE, W1–W7 molecules had values ranging from 0.99913 to 0.99967, with W6 having the highest value. The RE of WR molecule for electron (*λ*_−_) and hole (*λ*_+_) were 0.2017 eV and 0.2209 eV, respectively, and the range of RE for *λ*_−_ and *λ*_+_ of freshly proposed molecules was seen to be 0.1449–0.2031 eV and 0.1036–0.2278 eV, correspondingly. W1 and W5 seemed to be the best electron and hole carriers, respectively, among all, because of their smallest RE value for the electron (0.1449 eV) and hole (0.1036 eV), respectively. *V*_OC_ of all the studied small molecular acceptors was calculated by pairing them with PTB7-Th donor. The range *V*_OC_ and FF of newly proposed molecules was 0.95 to 1.51 eV and 0.8775 to 0.9144, respectively. W6 and W7 demonstrated the best results for the *V*_OC_ (1.48 eV and 1.51 eV), normalized *V*_OC_ (57.25 eV and 58.41 eV) and FF (0.9131 and 0.9144). Based on these findings, the use of these modified molecules in the production of OSCs with better photovoltaic characteristics should be considered in the future, with W1, W6, and W7 being the most prominent candidates amongst all.

## Author contributions

Ehsan Ullah Rashid: data collecting, processing, and assessment, as well as writing. Rasheed Ahmad Khera*: project management, data gathering, statistical analysis, graphics interpretation, and revision. Sahar Javaid Akram: curating data, detailed assessment, and the results interpretation. Javed Iqbal*: creating, securing funds, doing research, reviewing, and editing.

## Conflicts of interest

The Authors declare no conflict of interest.

## Supplementary Material
